# Endothelial senescence induced by PAI-1 promotes endometrial fibrosis

**DOI:** 10.1038/s41420-025-02377-0

**Published:** 2025-03-06

**Authors:** Jing Wu, Jie Wang, Zhongrui Pei, Yaru Zhu, Xier Zhang, Zihan Zhou, Chunying Ye, Minmin Song, Yali Hu, Pingping Xue, Guangfeng Zhao

**Affiliations:** 1 Department of Obstetrics and Gynecology, Nanjing Drum Tower Hospital Clinical College of Nanjing University of Chinese Medicine, Nanjing, China; 2 Department of Obstetrics and Gynecology, Nanjing Drum Tower Hospital, Affiliated Hospital of Medical School, Nanjing University, Nanjing, China; 3 Obstetrics and Gynaecology Hospital, Fudan University, Shanghai, China; 4 Department of Reproductive Medicine Center, Changzhou Maternal and Child Health Care Hospital, Changzhou Medical Center, Nanjing Medical University, Changzhou, China

**Keywords:** Mechanisms of disease, Reproductive disorders

## Abstract

Intrauterine adhesions (IUAs), also known as Asherman’s syndrome (AS), represent a significant cause of uterine infertility for which effective treatment remains elusive. The endometrium’s ability to regenerate cyclically depends heavily on the growth and regression of its blood vessels. However, trauma to the endometrial basal layer can disrupt the subepithelial capillary plexus, impeding regeneration. This damage results in the replacement of native cells with fibroblasts and myofibroblasts, ultimately leading to fibrosis. Endothelial cells (ECs) play a pivotal role in the vascular system, extending beyond their traditional barrier function. Through single-cell sequencing and experimental validation, we discovered that ECs undergo senescence in IUA patients, impairing angiogenesis and fostering stromal cell fibrosis. Further analysis revealed significant interactions between ECs and PAI-1+ stromal cells. PAI-1, derived from stromal cells, promotes EC senescence via the urokinase-type plasminogen activator receptor (uPAR). Notably, prior to fibrosis onset, TGF-β upregulates PAI-1 expression in stromal cells in a SMAD dependent manner. In an IUA mouse model, inhibiting PAI-1 mitigated EC senescence and endometrial fibrosis. Our findings underscore the crucial role of EC senescence in IUA pathogenesis, contributing to vascular reduction and fibrosis. Targeting PAI-1 represents a promising therapeutic strategy to suppress EC senescence and alleviate endometrial fibrosis, offering new insights into the treatment of IUAs.

## Introduction

Intrauterine adhesions (IUAs), also known as Asherman’s syndrome (AS), represent the leading cause of uterine infertility. This condition typically arises following trauma or inflammatory damage to the endometrium [1]. Currently, hysteroscopic adhesiolysis is the only recommended treatment, focusing on restoring the normal anatomical structure of the uterine cavity [2]. However, the recurrence rate of IUAs remains high, particularly in severe cases. The endometrium, with its unique cyclic tissue regeneration system, heavily depends on the cyclical growth and regression of its blood vessels [3, 4]. Trauma to the endometrial basal layer, can damage the subepithelial capillary plexus, thereby hindering endometrial regeneration [5]. As a result, native cells such as stromal cells, epithelial cells are replaced by myofibroblast cells, which produce excessive amounts of extracellular matrix leading to fibrosis. These cells produce excessive amounts of extracellular matrix, including collagen fibrils, leading to fibrosis [6, 7]. In densely fibrotic areas, the endometrium becomes thin and atrophic, with inactive glands and poorly vascularized stroma, making the environment not conducive to embryo implantation [8, 9]. While these pathological changes are well-documented, the underlying molecular mechanisms remain largely unknown.

Endothelial cells (ECs) are a dynamic component of the vascular system, extending their roles beyond the traditional barrier function [10]. Dysfunction of ECs is closely linked to fibrosis [11]. Specifically, ECs can undergo endothelial-to-mesenchymal transition (EndMT), leading to the accumulation of myofibroblasts [12, 13]. In addition, ECs release a variety of paracrine factors, such as angiocrine factors, that regulate the homeostasis, self-renewal, and differentiation of resident stem and progenitor cells, thereby modulating the fibrogenic response [14, 15]. ECs also can interact with other cells through adhesion molecules, chemokines, and exosomes, stimulating the production of pro-fibrotic cytokines and growth factors [16, 17]. Moreover, ECs play a crucial role in regulating vascular permeability and blood flow, which can influence the delivery of inflammatory and fibrogenic mediators to affected tissues [18].

The association between endothelial cell senescence and fibrosis is also receiving increasing attention. It is reported that the senescence of ECs triggers the development of liver fibrosis. Inhibiting thrombomodulin (THBD) signaling, which accelerates EC senescence in the liver, can effectively eliminate senescent cells and restore tissue homeostasis [19]. In the lung, age-related endothelial cell dysfunction contributes to progressive fibrosis, with activated cell states characterized by hypoxia, glycolysis, and persistent YAP/TAZ activity in aged mice [20]. However, the impact of endothelial senescence in the endometrium and its regulatory mechanisms remain unknown. This study aims to explore the effects of endometrial endothelial cell senescence on angiogenesis and fibrosis, as well as the mechanisms underlying endothelial cell senescence in IUA patients.

## Results

### Endometrial endothelial cells in IUA patients undergo senescence

**Fig. 1:**
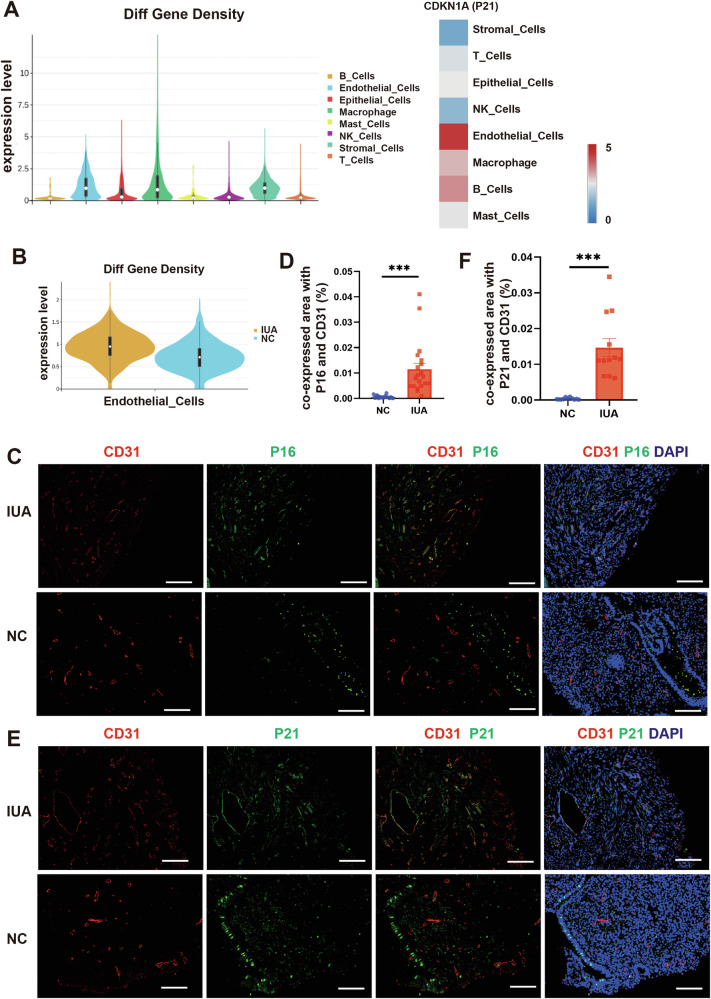
Endometrial endothelial cells in IUA patients undergo senescence. **A** Enrichment of senescent gene set in various cell types in endometrium (left), and CDKN1A enrichment condition (right). **B** Different expression of senescent genes between IUA patients and normal controls. Representative immunofluorescence images and quantized results for endothelial cells (CD31) and senescence, P16 (**C**, **D**) (*n* = 20) and P21 (**E**, **F**) (*n* = 12). All images are magnified × 200, and scale bar = 100 μm. Data are presented as the mean ± SEM, ***P < 0.001.

We first employed “SenMayo” [21], a senescent gene set, to analyze the senescence status of various cell subpopulations in the endometrium of both healthy individuals and patients with intrauterine adhesions (IUA). The results revealed that, compared to other clusters, this gene set demonstrated enrichment specifically in endothelial cells, macrophage and stromal cells. It is noteworthy that the typical senescence marker, such as CDKN1A (P21), exhibits a more pronounced enrichment in endothelial cells (Fig. 1A). Additionally, the expression levels of senescence-related genes (such as CDKN1A, CDKN2A, CD55, SEMA3F, CXCL8, CXCL3, CXCL2, and SPP1) in endometrial endothelial cells from IUA patients were notably higher than those in normal endometrial endothelial cells (Fig. 1B). Furthermore, we utilized immunofluorescence staining (IF) to investigate the expression of P21 and P16 (markers of senescence) as well as CD31 (a marker for endothelial cells) in endometria. The findings indicated that both P16 and P21 expressions were significantly elevated in the endometrial endothelial cells of IUA patients, suggesting the occurrence of senescence in these cells (Fig. 1C, E). Quantized results were showed in Fig. 1D, F.

### Senescent endothelial cells impair angiogenesis and promote fibrosis of stromal cells

**Fig. 2:**
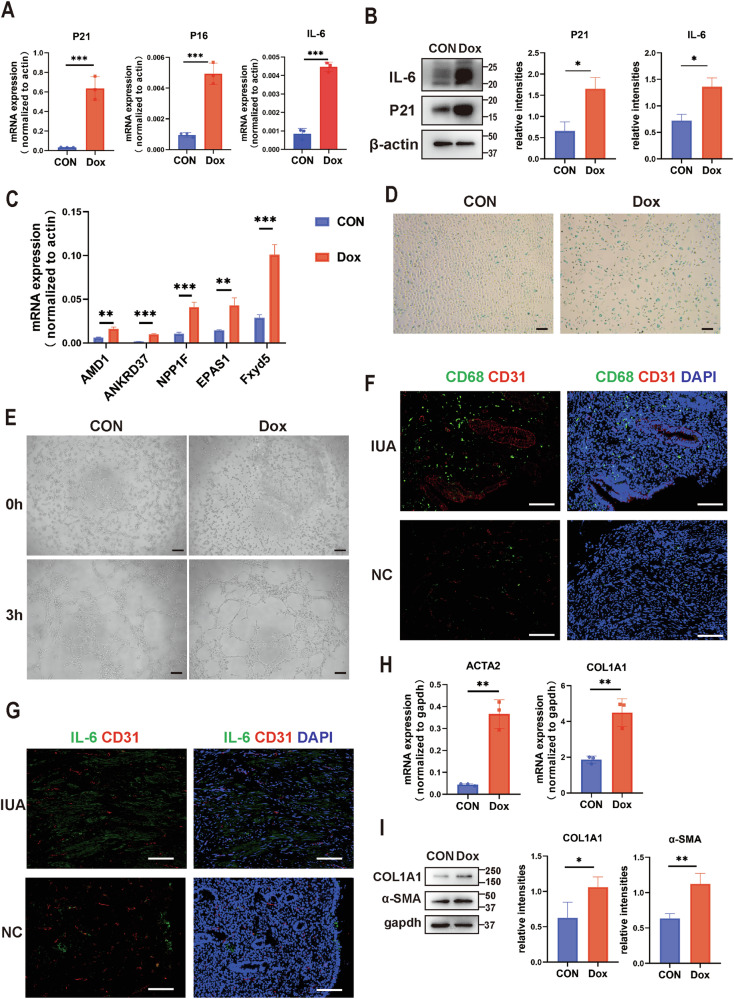
Senescent endothelial cells impair angiogenesis and promote fibrosis of stromal cells. **A** The mRNA expression levels of P21, P16, and IL6 in Dox+ HUVECs and Dox− HUVECs, relative to the control (β-actin) (*n* = 3). Data are presented as the mean ± SEM, ***P < 0.001. **B** Left: the relative protein level of P21 and IL-6 in HUVECs (*n* = 3). Right: the intension quantified by ImageJ, *P < 0.05. **C** The expression of endothelial active markers (AMD1, ANKRD37, NPP1F, EPAS1, Fxyd5) in response to Dox treatment (*n* = 3). **D** SA-β-gal staining (*n* = 3), scale bar = 100 μm. **E** Tube formation ability of HUVECs (*n* = 3), scale bar = 100 μm. **F** The localization of macrophages (CD68, green) and blood vessels (CD31, red) in endometria from normal control (*n* = 5) and IUA patients (*n* = 5), scale bar = 100 μm. **G** The localization of IL-6 (green) and blood vessels (CD31, red) in endometria from normal control (*n* = 5) and IUA patients (*n* = 5), scale bar = 100 μm. **H**, **I** The RNA and protein levels of ACTA2 and COL1A1 in ESCs treated with the medium of senescent HUVECs (*n* = 3). The intension of protein was quantified by ImageJ. Data are presented as the mean ± SEM, **P < 0.01, *P < 0.05.

To assess the effects of endothelial cell senescence, we initially developed a senescent model utilizing HUVECs treated with doxorubicin (Dox), a widely-used chemotherapeutic agent [22]. As the results demonstrate, we observed an increase in P21, P16, IL-6 (a canonical SASP marker), and endothelial cell activation marker genes (AMD1, ANKRD37, NPP1F, EPAS1, and FXYD5) in response to Dox treatment (Fig. 2A–C and Supplemental material). Additionally, these senescent ECs exhibited an elevated SA-β-gal staining (Fig. 2D) and lost their angiogenic capacity (Fig. 2E), indicating that senescence affects the basic functions of ECs and leads to their abnormal activation.

Studies have shown that active endothelial cells contribute to persistent fibrosis [20]. For instance, senescent ECs can secrete profibrotic mediators, pro-inflammatory cytokines, chemokines, and exosomes to recruit immune cells and trigger a cascade of changes [23]. We confirmed the presence of increased macrophages and inflammatory responses around vessels in patients with IUA (Fig. 2F, G). To further explore the effect of senescent ECs on fibrosis, we used the supernatant medium from senescent ECs to stimulate endometrial stromal cells (ESCs), which are the primary source of myofibroblasts. The results revealed that the supernatant from senescent ECs promoted the expression of high levels of fibrosis markers (ACTA2 and COL1A1) in stromal cells (Fig. 2H, I and Supplemental material). We also employed a replicative senescence model to verify the consequences of EC senescence [24]. The results further showed that with increasing passages of HUVECs, P21 expression rose (Supplementary Fig. 1A, B) and the ability of their culture supernatant to promote fibrosis in stromal cells became stronger (Supplementary Fig. 1C–F and Supplemental material).

### PAI-1 derived from endometrial stromal cells promotes senescence of ECs

**Fig. 3:**
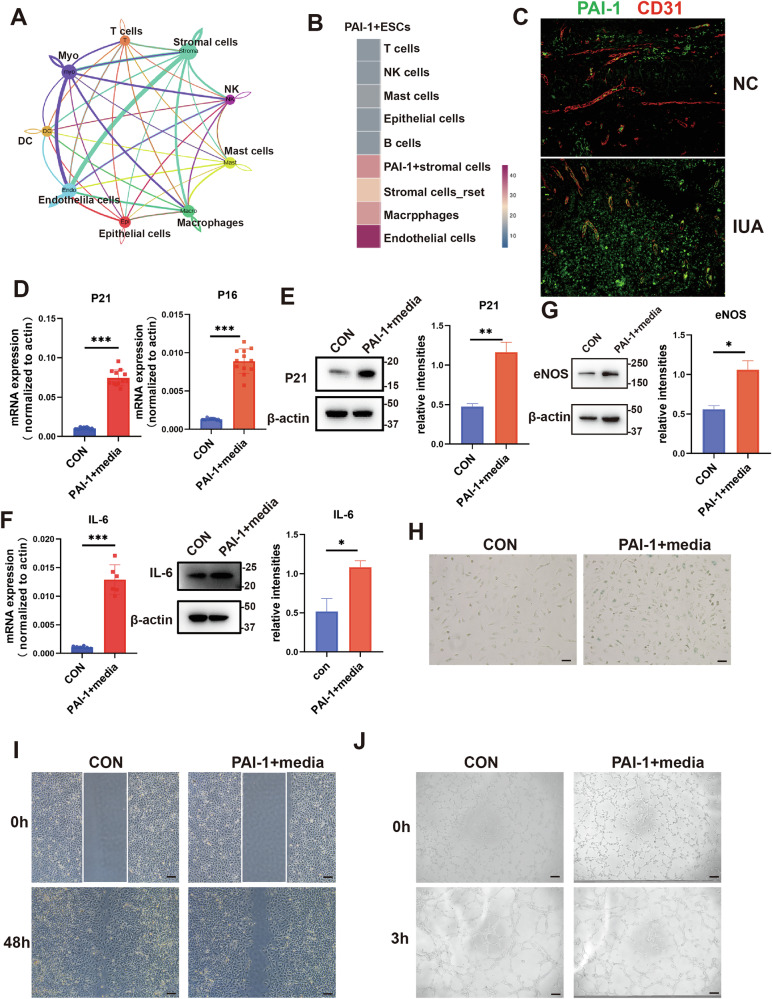
PAI-1 derived from endometrial stromal cells promotes senescence of ECs. **A** Analysis of interactions between various cells. **B** Analysis of interactions between PAI-1 + ESC and other various cells. **C** Immunofluorescence staining of PAI-1 (green) and vessels (red) in IUA patients (*n* = 5). scale bar = 100 μm. The supernatant from PAI-1+ ESCs was collected to stimulate HUVECs. **D**–**J**. Subsequently, the mRNA levels of P16 and P21 were analyzed by qPCR (*n* = 12) (**D**); the protein level of P21 was assessed by western blotting (*n* = 3) (**E**); the mRNA and protein levels of IL-6 were examined by qPCR (*n* = 6) and western blotting (*n* = 3), respectively (**F**); and the protein level of eNOS was determined by western blotting (*n* = 3) (**G**). SA-β-gal staining was observed (*n* = 3). scale bar = 100 μm (**H**), HUVECs migration was assayed (*n* = 3). scale bar = 100 μm (**I**), and tube formation was examined (*n* = 3) (**J**). scale bar = 100 μm.

To explore the causes leading to endothelial senescence, we used CellChat to analyze the interactions between endothelial cells and other cells in the endometrium of patients with IUA. The results showed that the interaction between endothelial cells and stromal cells was the most significant (Fig. 3A). Subsequently, we conducted further subpopulation analysis on stromal cells. As shown in the Supplementary Fig. 2A, stromal cells can be divided into ten subpopulations. Among of them, the group 3, which highly expresses SERPINE1(PAI-1), was significantly increased in the endometrium of IUA patients (Supplementary Fig. 2A).

Further CellChat analysis revealed that PAI-1+ESCs also exhibited a significant reaction to endothelial cells (Fig. 3B). Our immunohistochemical results also confirmed the high expression of PAI-1 in the endometrium of patients with IUA (Supplementary Fig. 2B), and PAI-1+ESCs were found to be located around CD31+ vessels (Fig. 3C). Based on the CellChat analysis results and co-location staining, we hypothesized that PAI-1 secreted by ESCs might affect the senescence of endothelial cells. Therefore, we collected the cell culture supernatants from ESCs with high PAI-1 expression to treat HUVECs. The results demonstrated that the supernatants with high PAI-1 expression induced a morphological transition of endothelial cells from cobblestone-like to flat (Supplementary Fig. 2C) and significantly promoted the expression of P21, P16, and IL-6 (Fig. 3D–F and Supplemental material), while inhibiting the expression of eNOS (Fig. 3G and Supplemental material). Concurrently, these endothelial cells exhibited a high positive area for SA-β-gal. Furthermore, these senescent cells displayed reduced capacity for migration and angiogenesis (Fig. 3H–J).

### PAI-1 promotes endothelial cell senescence through uPAR

**Fig. 4:**
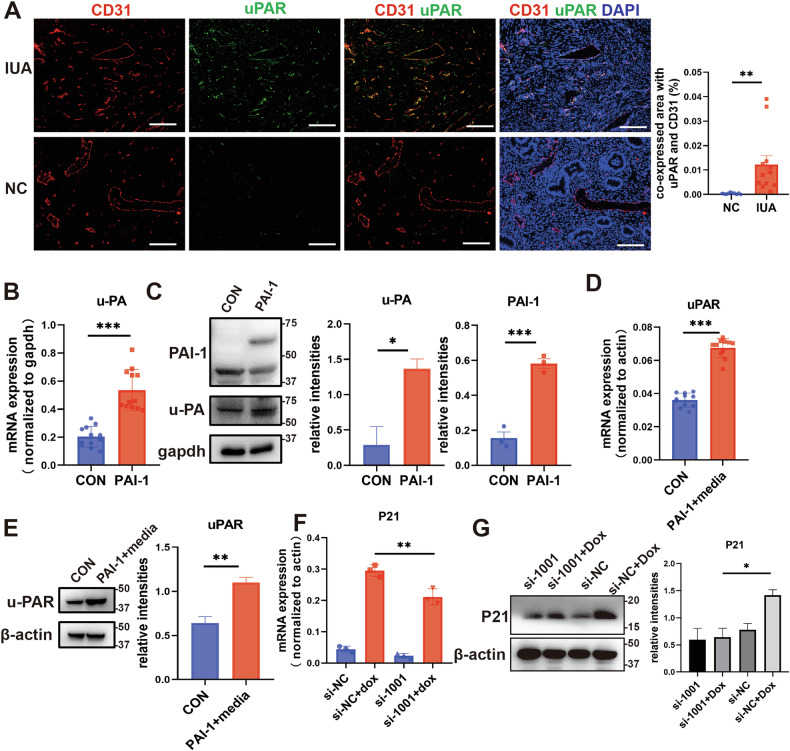
PAI-1 promotes endothelial cell senescence through uPAR. **A** Immunofluorescence staining of uPAR (green) and CD31 (red) in endometria from IUA patients (*n* = 12) and controls (*n* = 12). Quantized co-expression area (%) was showed at right. scale bar = 100 μm. **B**, **C** The mRNA (*n* = 12) and protein levels (*n* = 3) of uPA in PAI-1+ESCs. **D**, **E** The mRNA (*n* = 11) and protein levels (*n* = 3) of uPAR in HUVECs treated with PAI-1+ESCs supernatant. **F**, **G** The mRNA and protein levels of P21 (*n* = 3) in HUVECs treated with Dox and transfected with si-PLAUR.

Next, we delved into the molecular mechanisms underlying PAI-1’s promotion of endothelial senescence. We administered rhPAI-1 to treat HUVECs, yet rhPAI-1 failed to induce senescence in these cells (Supplementary Fig. 3A and Supplemental material). PAI-1 belongs to the fibrinolytic system [25], which also encompasses urokinase-type plasminogen activator (uPA) and reacts to urokinase-type plasminogen activator receptor (uPAR) [26]. Through their intricate interactions with extracellular matrix proteins, transmembrane receptors, and other intracellular signaling pathways, these components modulate cell migration, cell-matrix interactions, and signaling cascades [27]. Studies have documented an upregulation of uPAR in senescent cells [28]. The immunofluorescent staining results further substantiated this, revealing a larger co-staining area of CD31 with uPAR in patients with IUA compared to normal controls (Fig. 4A). Additionally, sc-RNA-seq analysis uncovered an elevation of PLAUR (uPAR) in the endometrium of IUA patients relative to normal controls (Supplementary Fig. 3B). Additionally, PAI-1+ ESCs expressed high levels of u-PA (Fig. 4B, C and Supplemental material). Consequently, we hypothesized that PAI-1, in collaboration with uPA, initiates endothelial senescence through uPAR, a receptor that has been shown to be upregulated in senescent cells [28]. To validate this, we treated HUVECs with supernatant derived from ESCs overexpressing PAI-1, leading to an increased expression of uPAR in these cells (Fig. 4D, E and Supplemental material). Furthermore, silencing uPAR with siRNA reversed the endothelial senescence induced by DOX (Fig. 4G, H and Supplemental material).

### TGF-β upregulates PAI-1 in SMAD dependent manner

We have proved that PAI-1 secreted by ESCs could promote endothelial senescence. PAI-1 is known as a plasminogen activator inhibitor both in physiological and pathologic circumstances to take part in fibrosis as well. Initially, we assessed the impact of PAI-1 overexpression on the fibrosis of ESCs themselves. The results indicated that PAI-1 overexpression did not affect the expression of ACTA2 and COL1A1 RNA and protein in ESCs (Supplementary Fig. 4A, B and Supplemental material), consistent with our sc-RNA-seq findings (Supplementary Fig. 4C). Additionally, we employed the cell-mediated ECM-degradation screening system [18] to further confirm that PAI-1 did not inhibit ECM degradation (Supplementary Fig. 4D). Collectively, these results suggested that PAI-1 itself may not directly participate in the fibrosis process in IUA. We further examined the impact of high PAI-1 expression on ESC senescence and found that ESCs did not undergo senescence when PAI-1 was highly expressed (Supplementary Fig. 5A, B and Supplemental material).

**Fig. 5:**
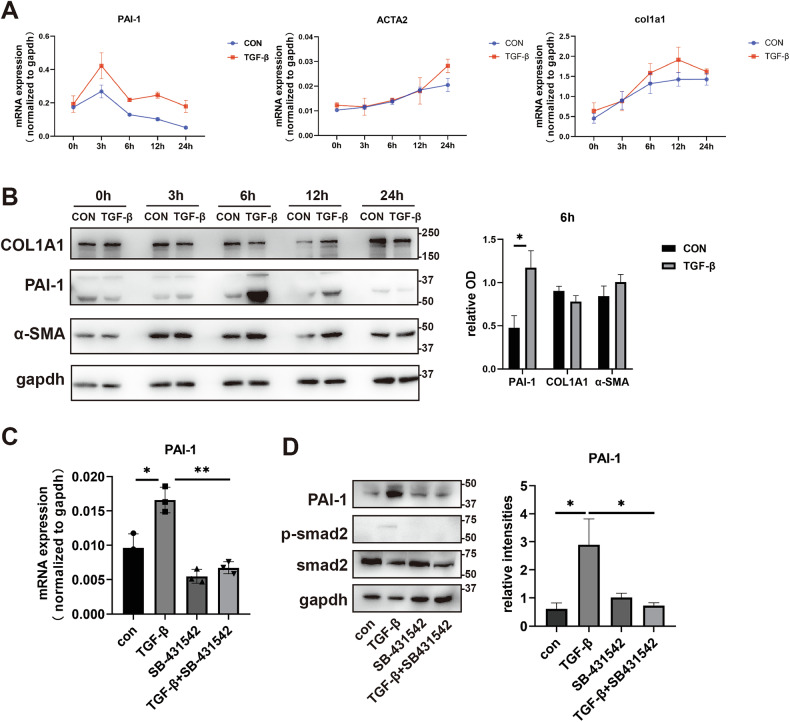
TGF-β upregulates PAI-1 in SMAD dependent manner. **A** The mRNA levels of PAI-1, ACTA2, and COL1A1 were examined by qPCR in ESCs after treatment with 10 ng/ml TGF-β for 3, 6, 12, 24 h respectively (*n* = 3). **B** The protein levels of PAI-1, ACTA2, and COL1A1 were tested by western blotting in ESCs after treatment with 10 ng/ml TGF-β for 6, 12, 24 h respectively (*n* = 3). **C** The mRNA level of PAI-1 were examined by qPCR in ESCs after treatment with 10 ng/ml TGF-β with or without SB-421542 (*n* = 3). **D** The protein levels of PAI-1, p-SMAD2, and SMAD2 were tested by western blotting in ESCs after treatment with 10 ng/ml TGF-β with or without SB-421542 (*n* = 3).

PAI-1 also was reported as a downstream of TGF-β, and TGF-β is a well-known molecule could induce transitions from fibroblasts to myofibroblasts. Therefore, to further exclude the relationship between PAI-1 and TGF-β in IUA, we used TGF-β to stimulate ESCs. Surprisedly, we found PAI-1 increased before ACTA2 and COL1A1 in the level of RNA (Fig. 5A) and protein (Fig. 5B and Supplemental material) after TGF-β treatment. We used SB-431542, an inhibitor of TGF-β to further investigate the mechanism of increased PAI-1. Results showed that TGF-β increased the level of PAI-1 according with the increased level of p-SMAD2, which is a canonical molecule of TGF-β signal. And after SB-431542 treatment, the expression of p-SMAD2 and PAI-1 were decreased (Fig. 5C, D and Supplemental material). It suggested that TGF-β could increase the level of PAI-1 via p-SMAD2.

### PAI-1 inhibitors inhibit endothelial senescence and endometrial fibrosis in mice

**Fig. 6:**
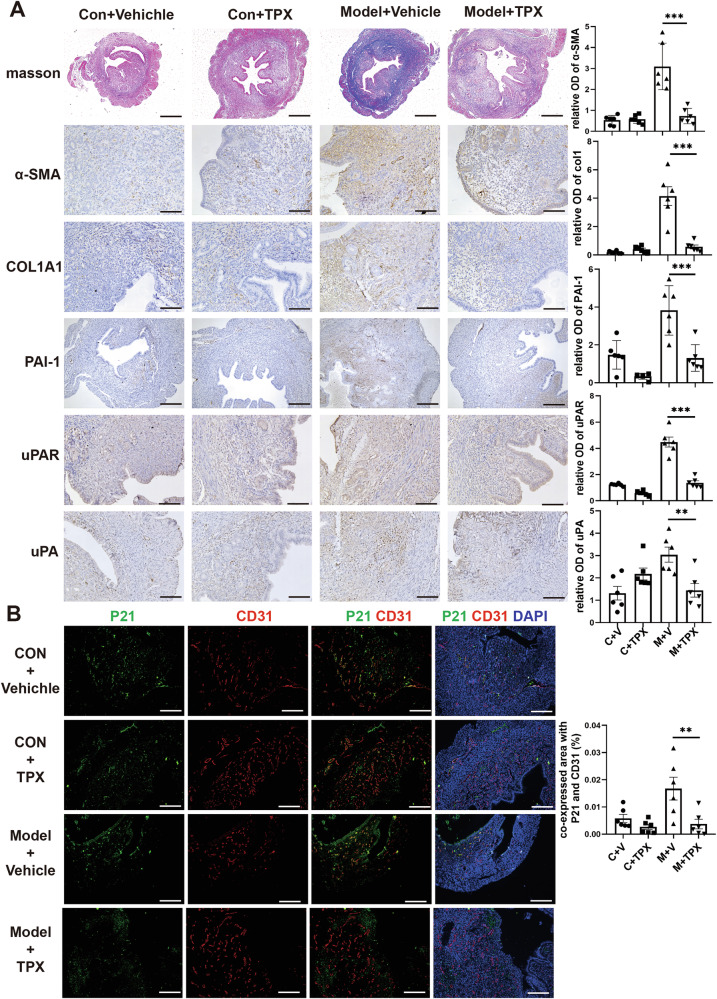
PAI-1 inhibitors can inhibit endothelial senescence and endometrial fibrosis in mice. **A** Masson’s trichrome staining and immunohistochemical staining for COL1A1, α-SMA, PAI-1, uPA, and uPAR were performed on endometrial samples from normal controls, both with and without TPX, as well as in the IUA mouse model, both with and without TPX (*n* = 6). scale bar = 100 μm. **B** Immunofluorescence staining was conducted to detect P21 (green) and CD31 (red) in endometrial samples from normal controls, both with and without TPX, and in the IUA mouse model, both with and without TPX (*n* = 6). scale bar = 100 μm. The quantized results analyzed by ImageJ showed on right, each bar represents the mean ± SEM. ANOVA test, **p < 0.001, ***p < 0.0001.

To gain a deeper understanding of the role of PAI-1-induced endothelial senescence in endometrial fibrosis in vivo, we established a dual-damage-induced IUA-like mouse model as previously described [6]. Our results revealed that the endometrium of the IUA model mice exhibited fibrosis, characterized by increased expression of α-SMA and Collagen I, as well as enhanced Masson staining (Fig. 6A). This was accompanied by elevated expression of PAI-1, uPA, and uPAR, and endothelial cell senescence, evidenced by increased p21 expression, consistent with clinical samples (Fig. 6B). We treated the IUA mice with Tiplaxtinin (TPX), an inhibitor of PAI-1. The results showed that TPX notably decreased the expression of PAI-1, uPA, and uPAR, and effectively alleviated endothelial senescence, as evidenced by the reduced expression of p21 (Fig. 6A, B). Furthermore, fibrosis was alleviated, with reduced expression of α -SMA and Collagen I, as well as diminished Masson staining (Fig. 6A).

## Discussion

Previous studies on endometrial fibrosis have largely concentrated on the contributions of epithelial cells, stromal cells, and immune cells [6, 7, 24], while endothelial cells have received scant attention. In our current research, single-cell sequencing analysis and experimental validation revealed that endothelial cells undergo senescence in patients with IUA. Furthermore, these senescent endothelial cells can subsequently trigger fibrosis in stromal cells. An analysis of cell interactions showed that endothelial cells interact most significantly with PAI-1+ stromal cells, which in turn promote endothelial cell senescence via PAI-1. Notably, PAI-1 can be induced by TGF-β and emerges prior to the onset of fibrotic changes. By inhibiting the PAI-1 pathway, we observed that endometrial fibrosis can be mitigated in vivo.

It is well-established that senescent cells can promote fibrosis in surrounding cells [29–32]. In our co-immunofluorescence staining results showed that the senescent markers are co-located with endothelial cells mostly, and the supernatant of senescent ECs can promote fibrosis in stromal cells, suggesting that senescent ECs can further facilitate fibrosis by releasing certain factors. It is known that senescent cells are characterized by SASP (inflammatory senescence-associated secretory phenotype), and factors like IL-6, IL-1, IL-8, and TNF-α are closely related to fibrosis [33, 34]. We observed a significant increase in IL-6 expression in senescent ECs in response to PAI-1+media secreted by ESCs. These results all showed that senescent cells are still active in metabolism. Excepted by secreted factors, the senescent cells accumulated instantaneously of can affect tissue homeostasis as well, which caused abnormal tissue remodeling [23].

Since 1977, PAI-1 has been acknowledged as a key regulator in fibrosis [35]. Nevertheless, our findings suggest that within the endometrium, PAI-1 does not directly contribute to stromal fibrosis or endothelial senescence. Rather, PAI-1 is more likely to be internalized along with uPA, rather than accumulated, to trigger endothelial senescence and subsequent stromal fibrosis in IUA in this study. The similar results have been proved in other study as well [26]. There are several studies have proved that PAI-1-uPA-uPAR, the complexes play important roles in different conditions. For example, PAI-1 can inhibits u-PA which induced chemotaxis via internalizing uPAR, to regulate cell migration [36]. uPAR is a famous receptor of u-PA. Active u-PA bound with uPAR on the cell surface, and the complex can be inhibited by PAI-1, resulting inactive PAI-1-uPA-uPAR complexes are internalized rapidly [37, 38]. Research has further supported this by showing that silencing the uPAR scavenger receptor in a renal fibrosis model leads to a significant accumulation of PAI-1 protein, ultimately resulting in severe fibrosis. Additionally, our study reveals that TGF-β stimulates an increase in PAI-1 expression in stromal cells via its typical signaling pathway before the onset of fibrosis via smads. Yuki et al. also has proved that smad2/3 which is induced by TGF-β could complexed with P53 in the PAI-1 promoter, consequently leading to relax the chromatin structure of PAI-1 and show active transcription [39]. These results indicate that PAI-1 is activated and expressed in stromal cells at an early stage, promoting endothelial senescence and ultimately leading to endometrial fibrosis.

**Fig. 7:**
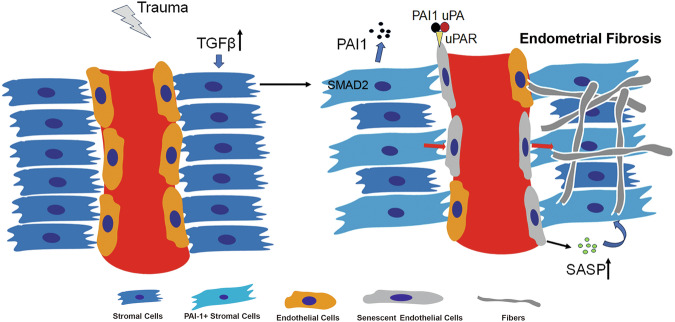
Schematic diagram illustrating how PAI-1 derived from ESCs promotes endothelial aging, leading to endometrial fibrosis. Trauma or infection elevates TGF-β in the endometrial microenvironment. TGF-β promotes the expression of PAI-1 in ESCs by activating SMAD2. PAI-1 synergizes with uPA to act on the uPAR receptor on endothelial cells, accelerating cellular senescence. Following endothelial cell senescence, the cells’ angiogenic capacity is inhibited, and they contribute to endometrial fibrosis by releasing factors such as SASP.

In summary, TGF-β promotes the expression of PAI-1 in ESCs by activating SMAD2. PAI-1 synergizes with uPA to act on the uPAR receptor on endothelial cells, accelerating cellular senescence. Following endothelial cell senescence, the cells’ angiogenic capacity is inhibited, and they contribute to endometrial fibrosis by releasing factors such as SASP (Fig. 7). Our results emphasize the crucial role of endothelial senescence in IUA, leading to vascular reduction and endometrial fibrosis. Inhibiting PAI-1 can effectively suppress endothelial senescence, thereby alleviating endometrial fibrosis. These findings provide fresh perspectives for the exploration of therapeutic strategies targeting IUA.

## Materials and methods

### Collection of endometrial tissues, and umbilical cords tissue

The Ethics Committee of Nanjing Drum Tower Hospital approved the collection of endometrial tissues (No. 2012022) and umbilical cords (No. 2021-214-01), and all participants provided written informed consents. We obtained the proliferative phase endometrium of IUA patients and normal controls through anonymized human endometrial biopsies. The late proliferative phase was defined by ultrasound measurements (15–17 mm) and low progesterone levels [40]. Patients diagnosed with severe IUA were identified based on hysteroscopic evaluation according to the criteria recommended by the American Fertility Society [41]. The control group consists of individuals with normal endometrium and ovaries, as confirmed by hysteroscopy and ultrasonography during the infertility screening process. Ten patients with IUA and 10 normal endometrial tissues were used for immunohistochemical analysis, while 5 normal endometrial tissues were used to isolate endometrial stromal cells (ESCs). Additionally, umbilical cords from healthy individuals (*n* = 20) who delivered at Nanjing Drum Tower Hospital were collected to isolate primary human umbilical vein endothelial cells (HUVECs).

### Single-cell RNA-seq data processing

The methodology utilized in this study aligns with our previous report [42]. Briefly, endometrial samples were washed with PBS, dissected into small fragments, and digested with 0.1% trypsin for 8 min, followed by treatment with 0.8 mg/ml Collagenase Type I for 60 min at 37 °C in a 5% CO_2_ atmosphere. The liberated cells were filtered, centrifuged, and treated with red blood cell lysis buffer prior to resuspension in PBS for single-cell 3’ cDNA library preparation. Cells expressing fewer than 200 genes or with mitochondrial gene content exceeding 15% were excluded from further analysis. Single-cell encapsulation, cDNA library synthesis, and RNA sequencing were conducted by Gene Denovo. The data were aligned to the human genome (GRCh38) using the STAR algorithm, and the unique molecular identifier count matrix was processed using the Seurat toolkit for normalization and log-transformation.

### Cell culture and treatment

For the preparation of HUVECs, the umbilical cords were trimmed to remove the clamped sections and any hematomas. Using a gavage needle, the umbilical veins were washed with PBS, followed by the injection of collagenase type 1 (1 mg/ml) into the veins for a duration of 10–15 min. After digestion, the digestive medium was collected, and the veins were subsequently washed with high-glucose DMEM (Wisent Inc., Canada) containing 10% fetal bovine serum (FBS; Gibco, USA). HUVECs were then cultured in Endothelial Cell Medium (ECM, ScienCell, catalog #1052) at 37 °C with 5% CO_2_. For treatment with Doxorubicin (Dox, MCE, HY-15142), HUVECs were incubated in ECM supplemented with 200 nM of Dox for 24 h. The supernatant collected from senescent HUVECs were used to culture ESCs for 24 h. To silence the PLAUR gene (GenePharma, China), HUVECs were transfected using Lipofectamine 3000 (Invitrogen, USA) according to the manufacturer’s instructions for 12 h prior to Dox treatment. The siRNA sequence used for PLAUR silencing was: ACCAUCACCCUGCUAAUGATT. The recombinant human PAI1 (R&D Systems, cat: 1786-PI-010, USA) was dissolved in the culture medium and subsequently administered to the cells for a duration of 60 h.

For the preparation of endometrial stromal cells (ESCs) [6, 43], fresh endometrial tissues were cut into small fragments and digested with a mixture of collagenase type I (Sigma, C2674, USA), hyaluronidase (Sigma, H3506, USA), and DNase (Roche, 10104159001, Switzerland) at 37 °C. The digested tissues were then filtered through a 40-μm pore filter (BD Falcon, 352340, USA) to isolate stromal cells, which were subsequently inoculated into DMEM/F12 (Biochannel, China) containing 10% FBS (Biochannel, China). Primary ESCs were transfected with Ad-serpine1, resulting in cells designated as PAI-1+ ESCs and the supernatant were collected as PAI-1+media after 24 h of culture which was used to treat HUVECs. The serpine1 overexpression plasmid was synthesized based on the sequence NM_001386460.1 (Generay, China) using pcDNA3.1 as the vector. For the TGF-β treatment group, cells were stimulated with 10 ng/ml TGF-β (Novoprotein, CA59, China) and SB-431542 (MCE, CAT: HY10431, China) dissolved in the culture medium.

### RNA extraction and quantitative real-time PCR (qRT-PCR)

Total RNA was isolated using TRNzol reagent (Tiangen, DP424, China) and measured by Nanodrop (Thermo Fisher Scientific, USA). 1 ug RNA was reverse transcribed into cDNA by Synthesis Kit (Vazyme, R323-01, China). The qRT-PCR was performed by ChamQ SYBR® qPCR Master Mix (Vazyme, Q321-02, China) in the LightCycler 480 machine (Roche, Switzerland) and gapdh or β-actin served as housekeeping genes. The relative expression of the genes was determined by the 2 − ΔCt. All primer sequences were listed in Supplement Table S1.

### Western blotting analysis

Proteins of cells were extracted using mixture of lysis buffer (Biosharp, BL509A), protease inhibitor cocktail (MedChemExpress, HY-K0010, China), and phosphatase inhibitor cocktail II (MedChemExpress, HY-K0022, China). Proteins concentrations were measured by the Pierce BCA protein reagent kit (Thermo Scientific, 23,225, USA). Next, 20 ug of protein were separated by 10% or 12.5% SDS-PAGE gel, and then transferred onto PVDF membranes (Bio-Rad, USA). The PVDF was incubated in 5% defatted milk (Bio-Rad, USA) for 1 h, followed by incubation with primary antibodies. After washed three times with TBST (Solarbio, T1082, China), it was hybridized with secondary antibodies for 1 h. The signals were visualized by ECL solution (Bio-Rad, USA) and quantified by analyzing the integrated density normalized to the level of an internal reference using ImageJ. The antibodies were listed in Supplement Table S2.

### Immunohistochemistry and Immunofluorescence staining

Human and mouse endometrial tissues were fixed in 4% paraformaldehyde overnight and then embedded in paraffin. The paraffin-embedded tissues were sectioned at 2 μm thickness and mounted on polylysine-coated glass slides. The paraffin-embedded tissues were cut into 5-μm-thick slices and stained with Masson’s Trichrome according to the kit instruction (Solarbio, China). For immunohistochemical analysis, following deparaffinization, rehydration, and blockage of endogenous peroxidase activity with 3% H_2_O_2_, antigen retrieval was performed. The tissues were then incubated with primary antibodies at 4 °C overnight. the tissues were washed with PBST and subsequently incubated with HRP-conjugated secondary antibodies (Typng, China) for 8 min. The antigen signals were visualized using DAB (Typng, TPB13, China) and imaged under a microscope (DMi8, Leica, Germany). For immunofluorescence analysis, after washing with PBST, light-protected secondary antibodies were added and incubated at room temperature for 1 h. Nuclei were labeled with DAPI (Abcam, 104139, USA). Images were captured using a microscope. The positive staining was confirmed in a blinded manner by two independent observers. The primary antibodies were listed in Supplement Table S2.

### Tube formation assay

HUVECs were collected and suspended in either PAI-1 + ESC-conditioned media or control ESC-conditioned media. Subsequently, 3 × 105 HUVECs were plated onto 96-well plates coated with Matrigel (Corning, 354230, USA) and incubated for 3 h. Tubular structure images were captured using a microscope.

### Scratch assay

HUVECs were seeded into 6-well plates. Once the cells reached 80% confluence, a scratch was made using a 1 ml pipette tip. The cell culture medium was then replaced with either PAI-1 + ESC-conditioned media or control ESC-conditioned media. The migration distance was measured after 48 h.

### SA-β-GAL staining

The activity of senescence-associated β-galactosidase (SA-β-GAL) was detected using a senescence β-GAL staining kit, following the manufacturer’s protocol (Beyotime, C0602, China). HUVECs subjected to different treatments were fixed in 4% paraformaldehyde for 10 min and then stained in an X-gal solution at 37 °C for 24 h, protected from light. Images of the stained cells were captured using an inverted microscope.

### Animal model

All animal experiments were approved by the Institutional Animal Care and Use Committee at Nanjing Drum Tower Hospital (DWSY-24091547). The left uterus (*n* = 12) of female C57BL/6J mice aged 6–8 weeks was used to establish IUA-like models as previously reported [6]. Dual-damage was induced by mechanical impairment (scraping 50 times) and LPS treatment for 5 min (1 mg/kg; Sigma, L2880, USA). The right uterus (*n* = 12) served as the sham group. Half of the mice (*n* = 6) were randomly injected with TPX (2.5 mg/ml, MCE, HY-15253, China) for a week, while the other half (*n* = 6) were injected with vehicle. The mice were euthanized, and their uteri were collected for further analysis in a blinded manner.

### Statistics

Statistics were analyzed using GraphPad Prism (Version 5.0, USA) and were presented as mean ± SEM. Differences were analyzed using unpaired *t*-test to compare two groups or the one-way analysis of variance (ANOVA) to compare multiple groups. The significant differences were defined as *P* < 0.05.

## Supplementary information

Supplementary figures

Full and uncropped western blots.

Table S1.

Table S2.

## Data Availability

All datasets are available from the corresponding author on reasonable request.
