# Supplementary material for: Endothelial senescence induced by PAI-1 promotes endometrial fibrosis
Source: Cell Death Discov. 2025 Mar 6;11:89. doi: 10.1038/s41420-025-02377-0 (PMC11885584; doi:10.1038/s41420-025-02377-0)
Supplement: Supplementary file 1 — Supplementary figures [file 41420_2025_2377_MOESM1_ESM.docx]

**Endothelial senescence induced by PAI-1 promotes endometrial fibrosis**

Jing Wu^1^, Jie Wang^1^, Zhongrui Pei^1^, Yaru Zhu^1^, Xier Zhang^1^, Zihan Zhou^2^, Chunying Ye^1^, Minmin Song^4^, Yali Hu^2, ✉^, Pingping Xue^3,^ ^✉^, Guangfeng Zhao^1,2,^ ^✉^

1 Department of Obstetrics and Gynecology, Nanjing Drum Tower Hospital Clinical College of Nanjing University of Chinese Medicine, Nanjing, China

2 Department of Obstetrics and Gynecology, Nanjing Drum Tower Hospital, Affiliated Hospital of Medical School, Nanjing University, Nanjing, China

3 Department of Reproductive Medicine Center, Changzhou Maternal and Child Health Care Hospital, Changzhou Medical Center, Nanjing Medical University, Changzhou, 213000, China

4 Obstetrics and Gynaecology Hospital, Fudan University, Shanghai, China

^✉^Corresponding authors: Guangfeng Zhao: [zhaoguangfeng@nju.edu.cn](mailto:zhaoguangfeng@nju.edu.cn); Pingping Xue: [13616118039@163.com](mailto:13616118039@163.com); Yali Hu: [yalihu@nju.edu.cn](mailto:yalihu@nju.edu.cn)

**Supplementary Figure 1. Senescent endothelial cells promote fibrosis of stromal cells.**


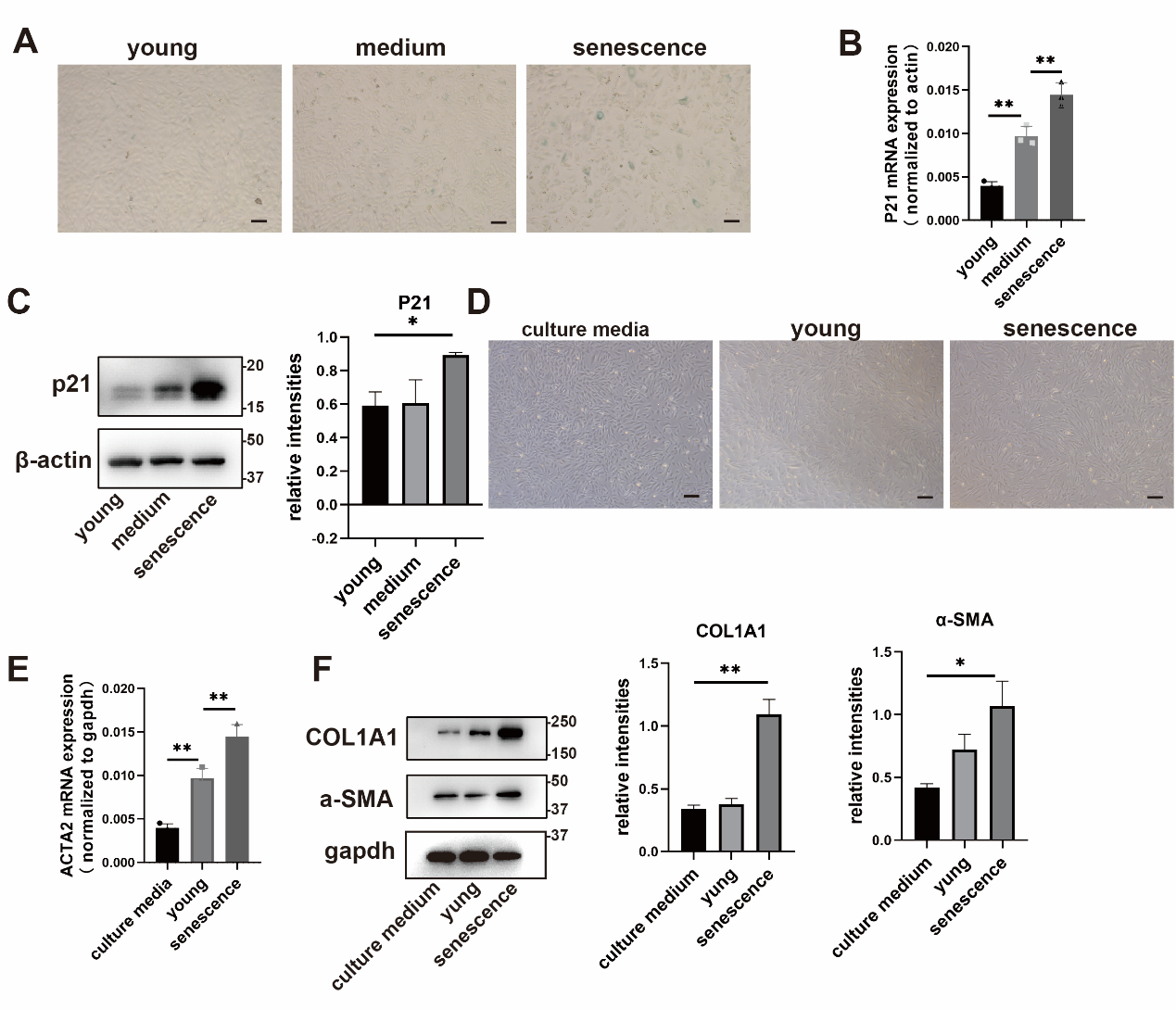


**Supplementary Figure 1** **Senescent endothelial cells promote fibrosis of stromal cells.** The 1st to 3rd passages of HUVECs are considered young cells; the 6th and 7th passages are medium cells; and those beyond the 10th passage are senescent cells. **A** SA-β-gal staining of HUVECs. scale bar=100μm. **B-C** mRNA and protein levels of P21in these HUVECs. **D** Morphological changes of ESCs after being treated with the supernatant from three different states of HUVECs for 48 hours. scale bar=100μm. **E-F** Fibrotic changes in RNA and protein levels of ESCs after being treated with the supernatant from three different states of HUVECs for 48 hours. Data are presented as the mean±SEM, **P＜0.01, *P＜0.05.

**Supplementary Figure 2 PAI-1 derived from endometrial stromal cells promotes senescence of ECs.**


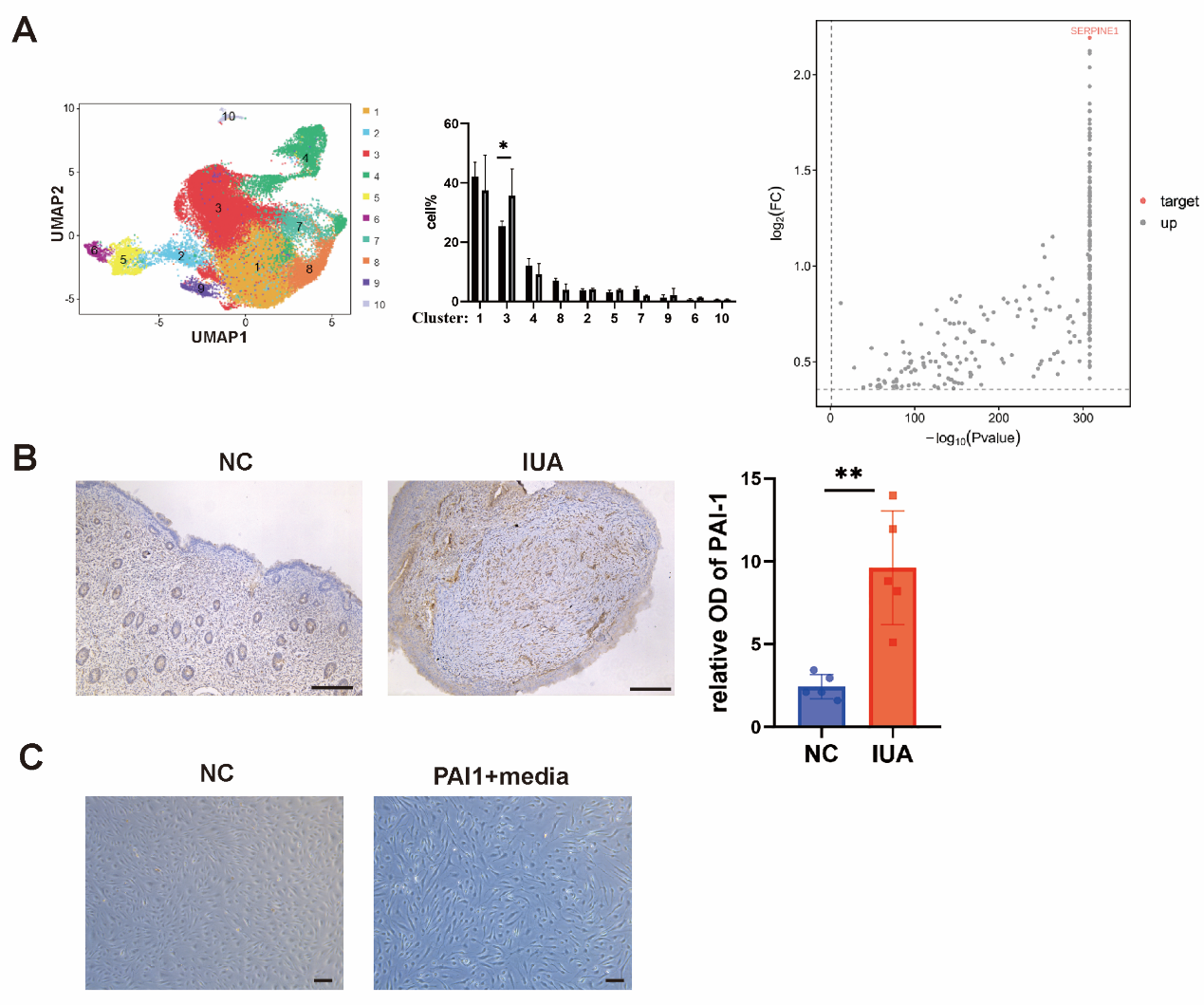
 **Supplementary Figure 2 PAI-1 derived from endometrial stromal cells promotes senescence of ECs.** **A** The ESCs were re-clustered into 10 subpopulations (left), and the cluster 3 exhibited significant increases in IUA patients (middle). Notably, these increases were associated with high expression of serpine1, which encodes the PAI-1 protein (right). **B** Immunohistochemistry staining PAI-1 in the endometria of IUA patients. scale bar=100μm. **C** Morphological changes of HUVECs after treatment with supernatant of PAI-1+ESCs. Scale bar=100μm. Data are presented as the mean±SEM, **P＜0.01, *P＜0.05.

**Supplementary Figure 3 PAI-1 promotes endothelial cell senescence through uPAR.**


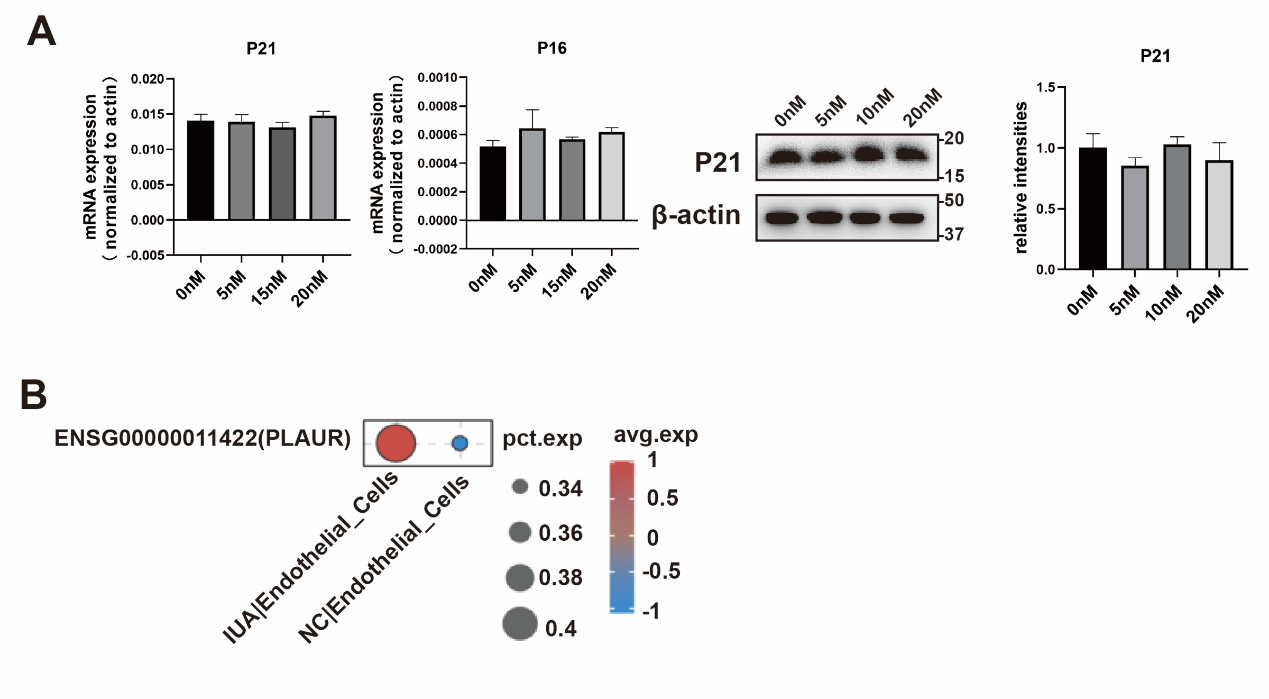
 **Supplementary Figure 3 PAI-1 promotes endothelial cell senescence through uPAR.** **A** HUVECs were treated with rhPAI-1 (0nM, 5nM, 10nM, and 20nM) for 60h, and then the expression of P21 and P16 was examined at both mRNA and protein levels. **B** The expression of PLAUR in endometrial endothelial cells from normal individuals and patients with IUA based on sc-RNA seq data.

**Supplementary Figure 4** **PAI-1 has no effects on senescence of ESCs.**


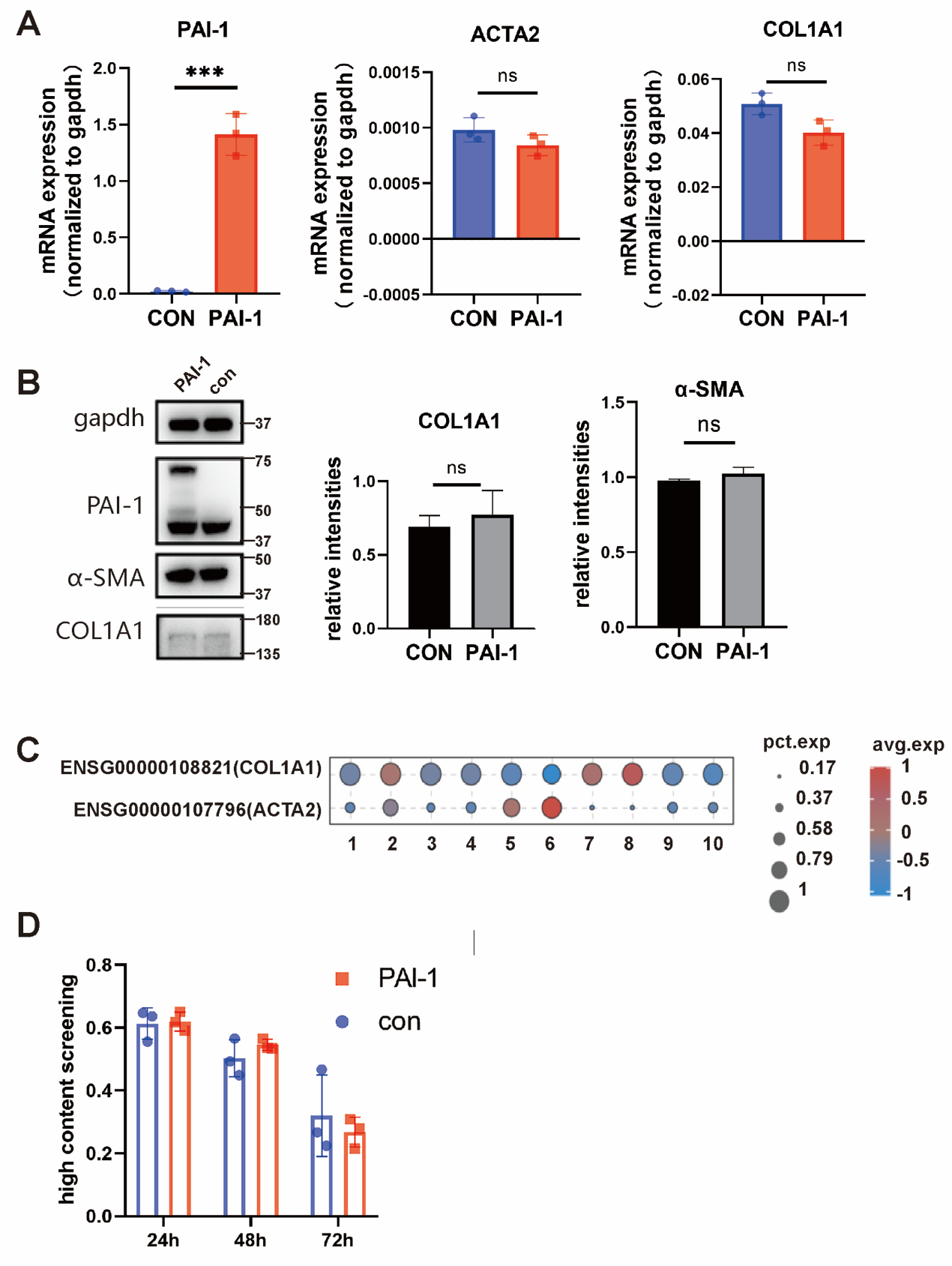
 **Supplementary Figure 4** **PAI-1 has no effects on senescence of ESCs.** The mRNA (**A**) and protein (**B**) levels of ACTA2 and COL1A1 in PAI-1+ESCs. **C** The expression of ACTA2 and COL1A1 in PAI-1+ESCs (cluster 3) based on sc-RNA seq. **D** Collagen degradation ability of PAI-1+ESCs characterized by CEDSS. Data are presented as the mean±SEM, ***P＜0.001; ns, P＞0.05.

**Supplementary Figure 5 PAI-1 has no effects on fibrosis of ESCs.**


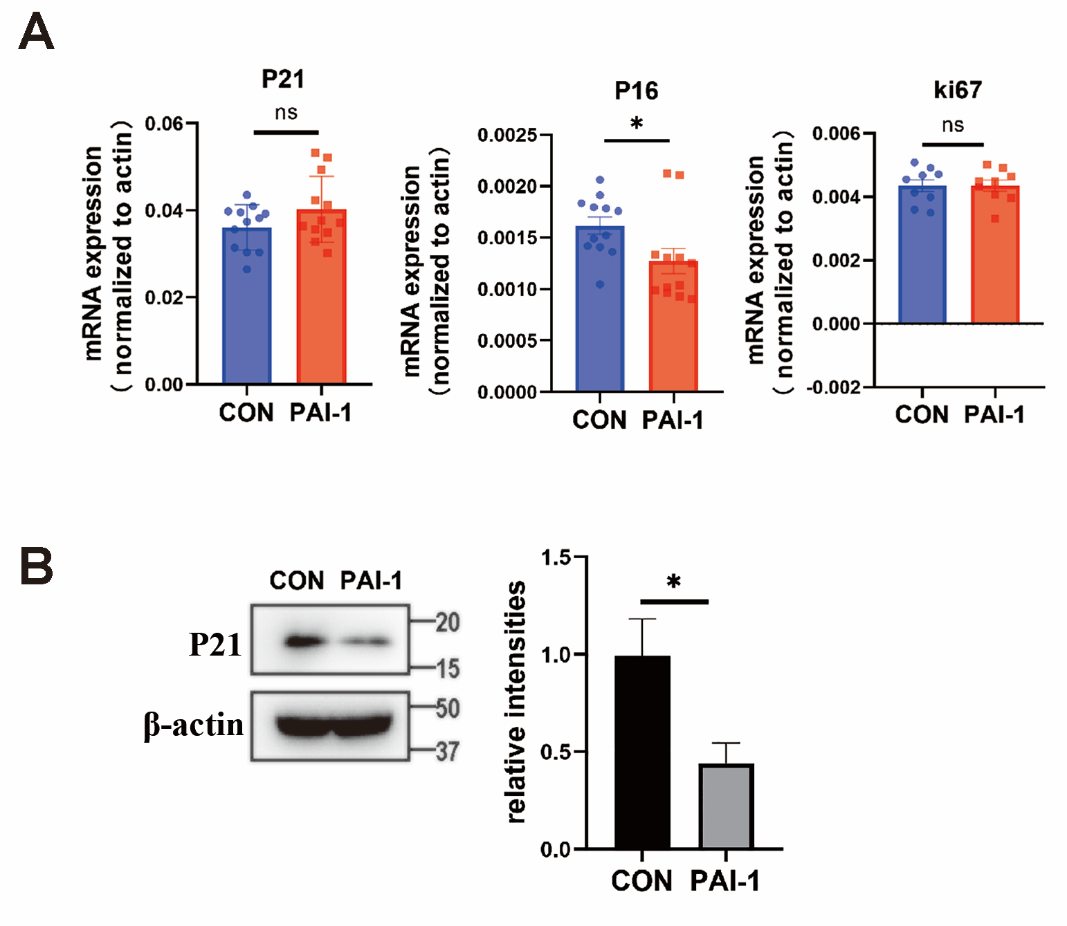


**Supplementary Figure 5 PAI-1 has no effects on fibrosis of ESCs.** **A** The mRNA levels of P21, P16 and Ki67 in PAI-1+ESCs. **B** The protein level of P21 in PAI-1+ESCs. Data are presented as the mean±SEM, *P＜0.05; ns, P＞0.05.
