# Supplementary figures and images for: Endothelial senescence induced by PAI-1 promotes endometrial fibrosis
Source: Cell Death Discov. 2025 Mar 6;11:89. doi: 10.1038/s41420-025-02377-0 (PMC11885584; doi:10.1038/s41420-025-02377-0)

**Supplemental material.**

Full and uncropped western blots.


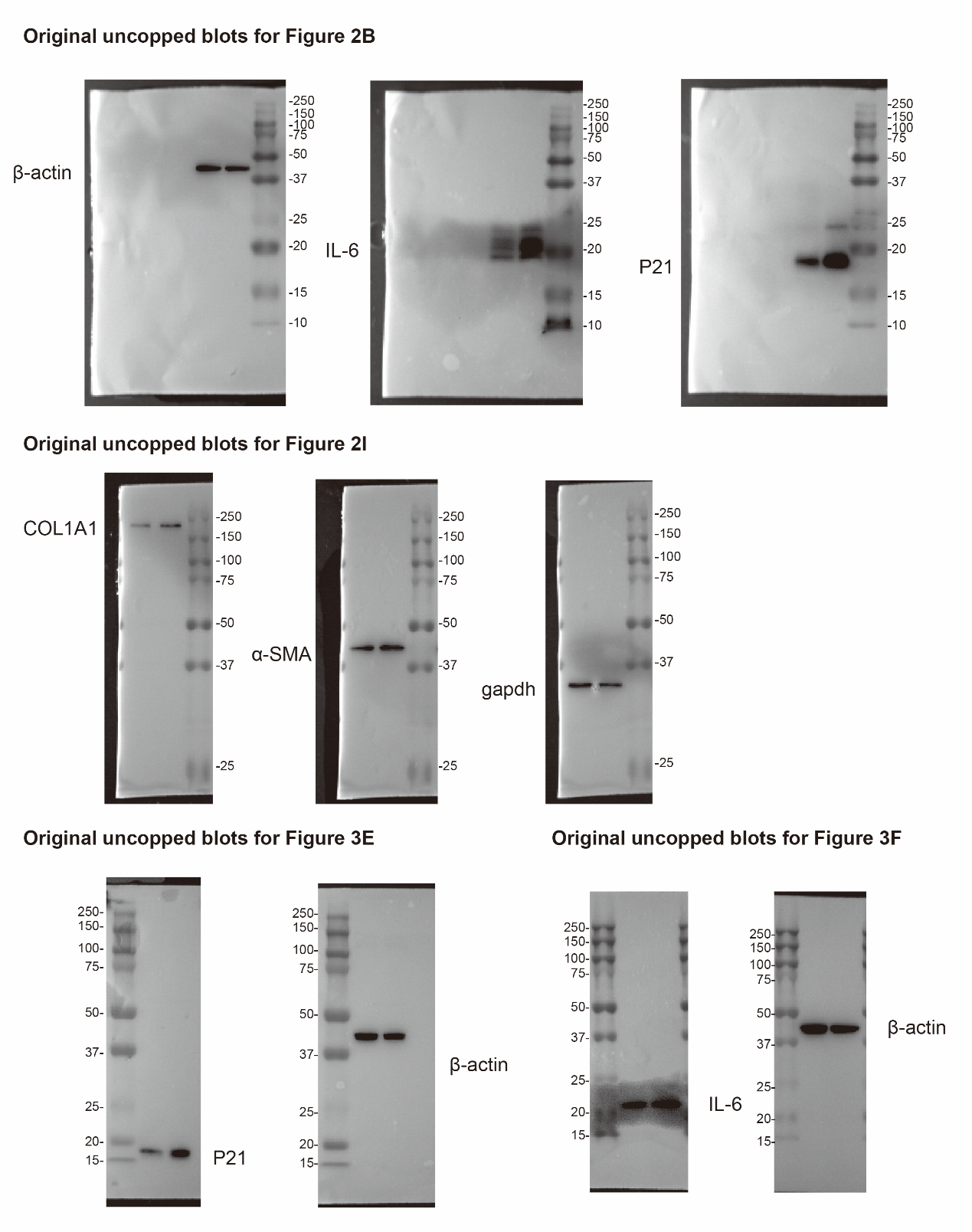

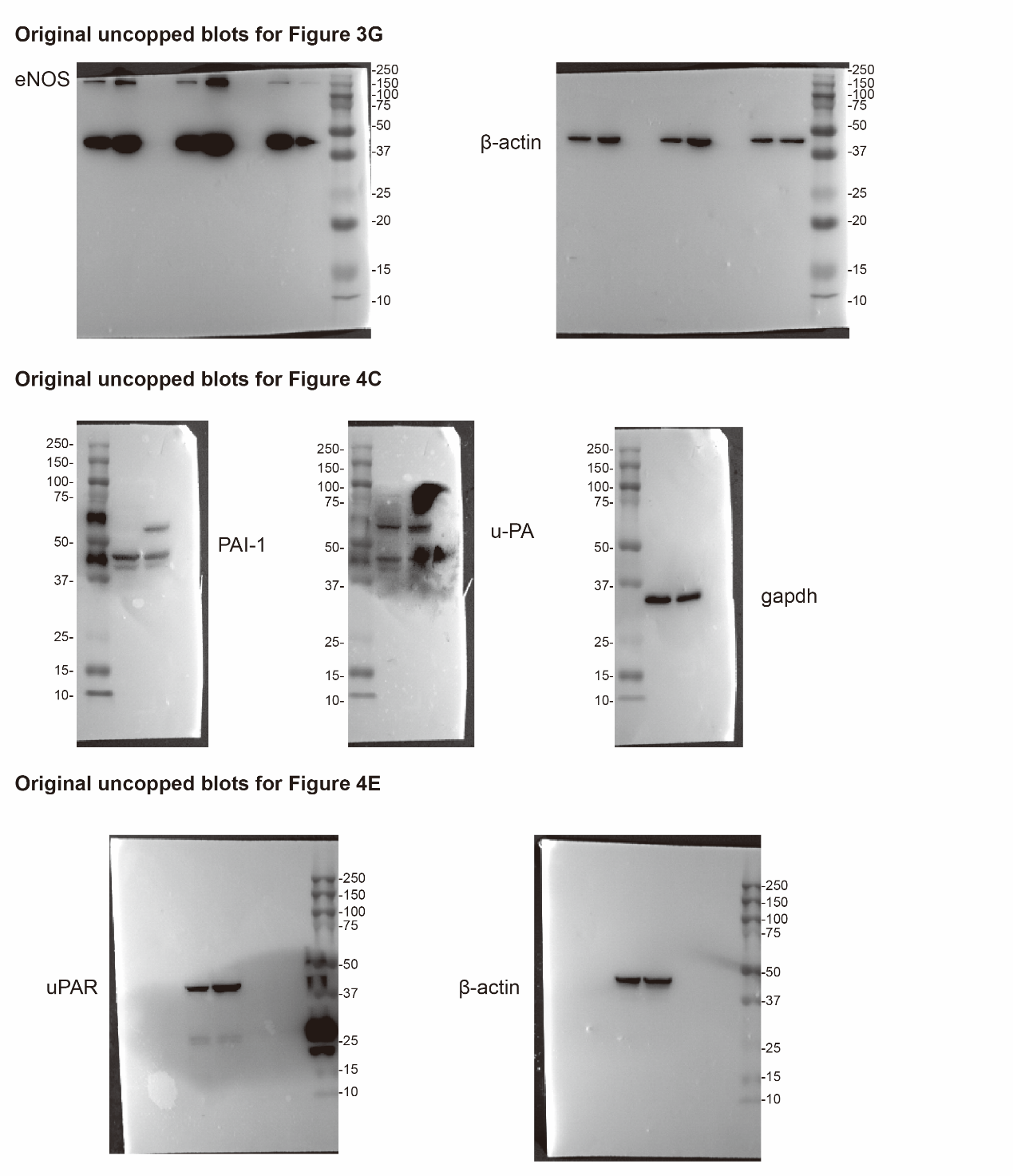

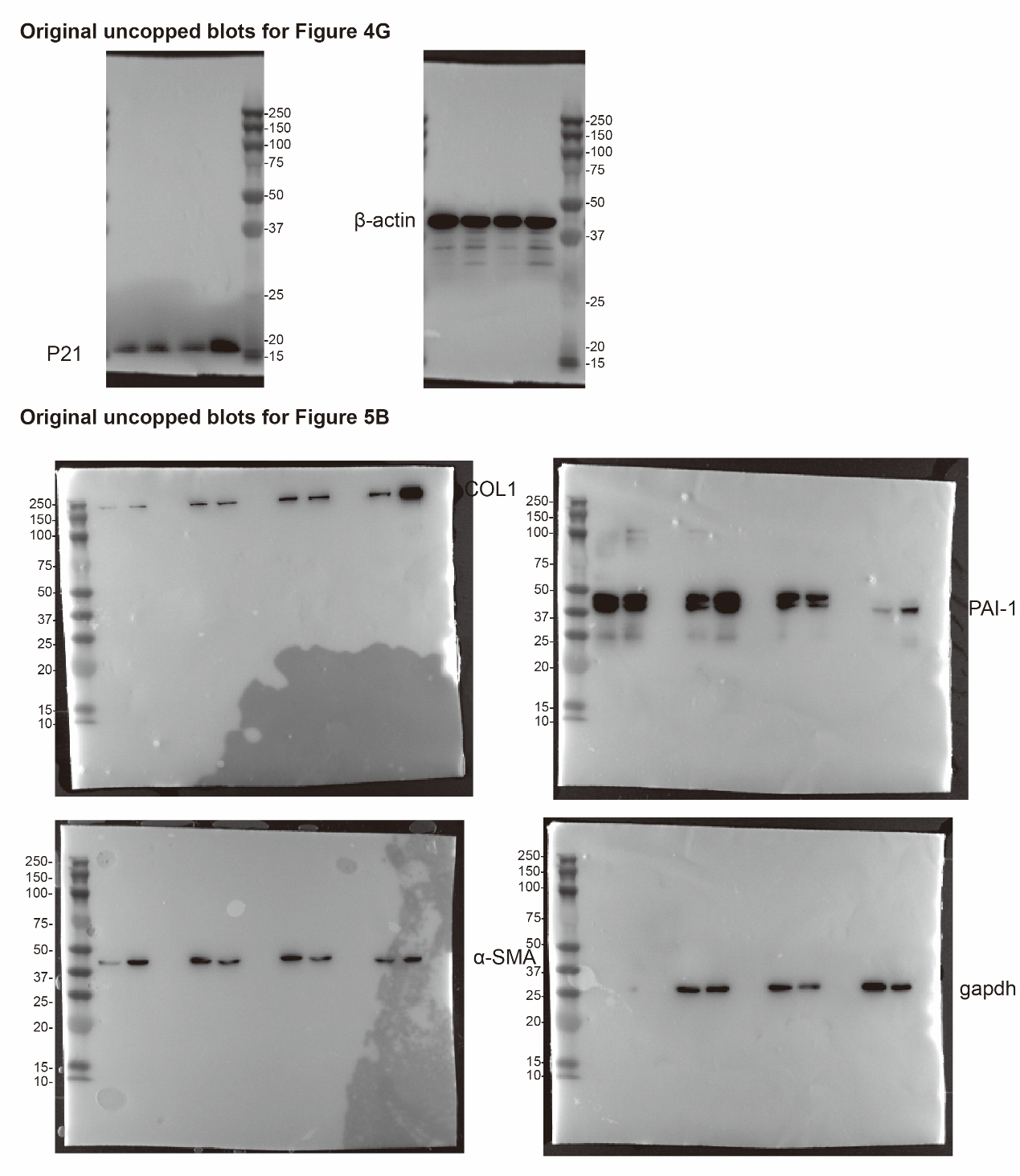


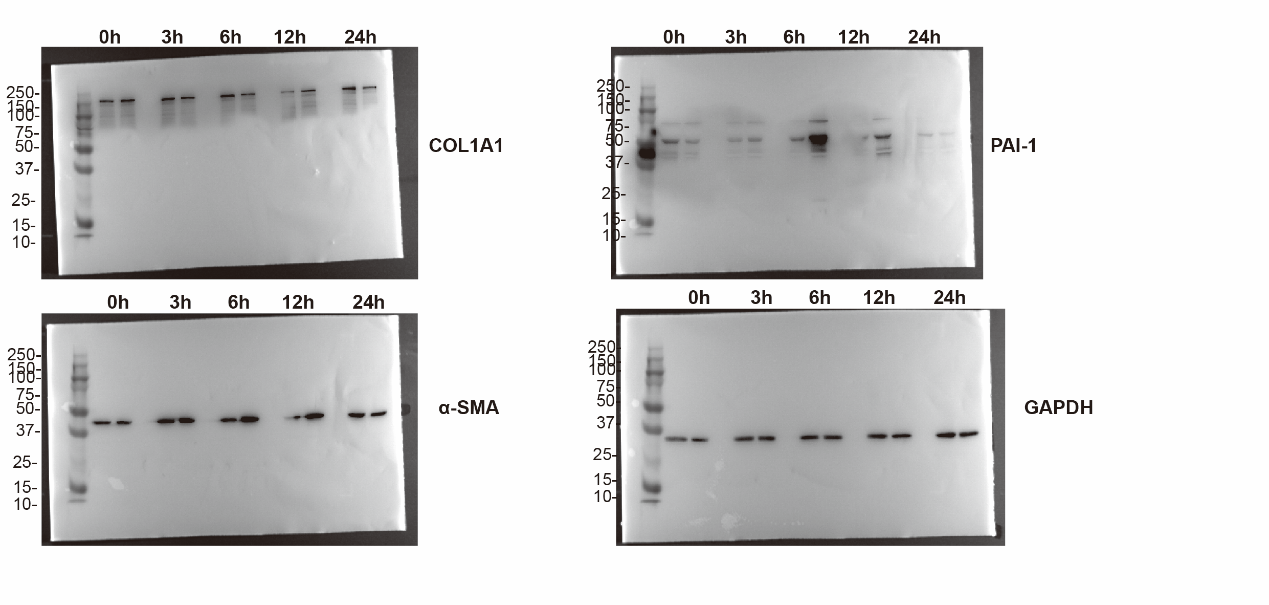


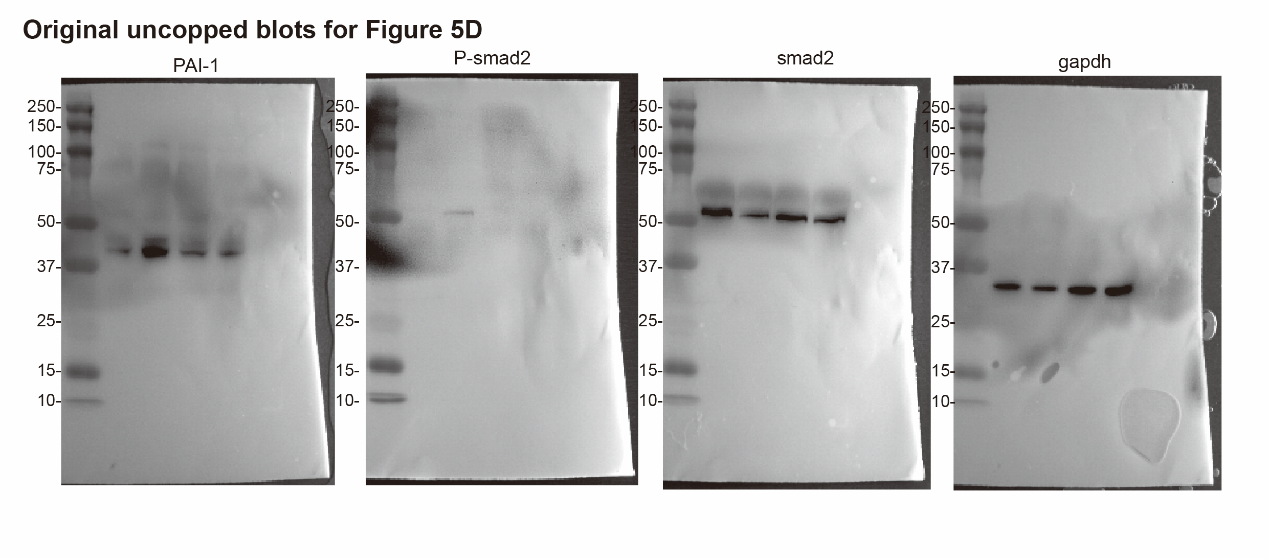

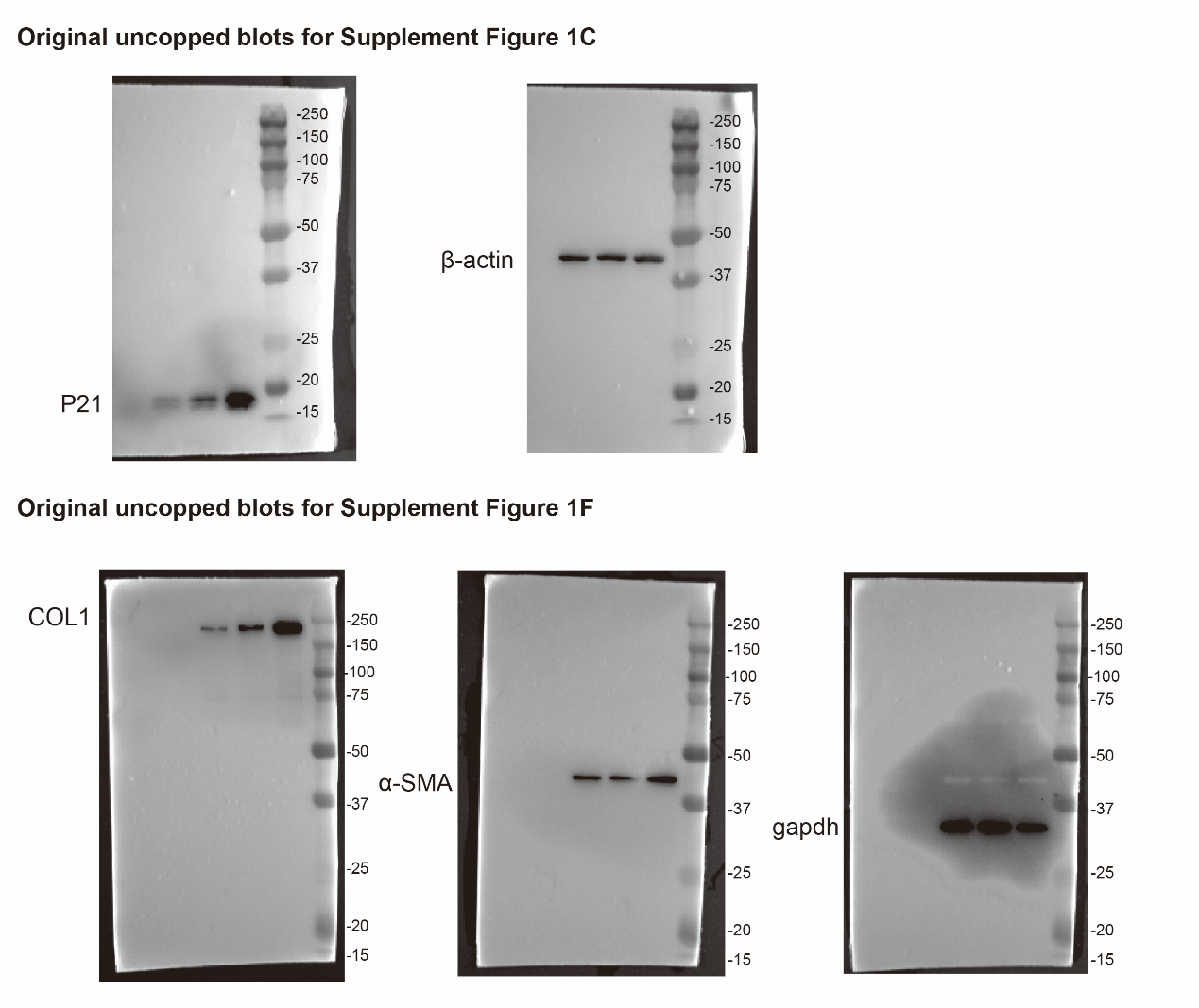

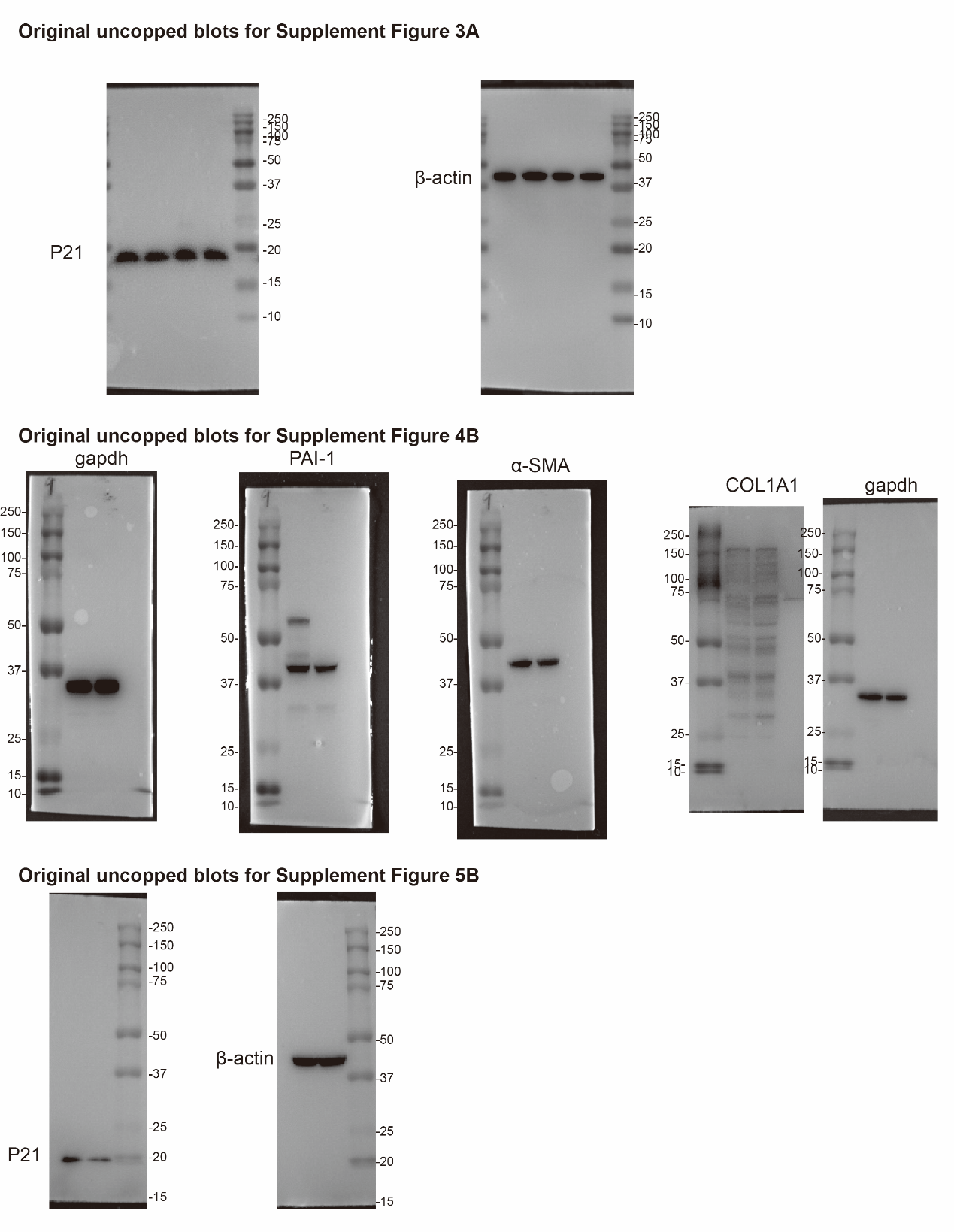

Supplement: Supplementary file 2 — Full and uncropped western blots. [file 41420_2025_2377_MOESM2_ESM.docx]
