# Supplementary material for: Endothelial senescence induced by PAI-1 promotes endometrial fibrosis
Source: Cell Death Discov. 2025 Mar 6;11:89. doi: 10.1038/s41420-025-02377-0 (PMC11885584; doi:10.1038/s41420-025-02377-0)
Supplement: Supplementary file 3 — Table S1. [file 41420_2025_2377_MOESM3_ESM.docx]

**Table S1: qPCR primer sequences**

| Gene | Forward | Reverse |
| --- | --- | --- |
| h-p16 | CAAGATCACGCAAAAACCTCTG | CGACCCTATACACGTTGAACTG |
| h-P21 | TGTCCGTCAGAACCCATGC | AAAGTCGAAGTTCCATCGCTC |
| h-IL-6 | TGAAAGCAGCAAAGAGGCACTG | TGAATCCAGATTGGAAGCATCC |
| h-AMD1 | AGTCGGGTAATCAGTCAGCCA | ACTCTCACGAGTGACATCCTTT |
| h-ANKRD37 | TTAGGAGAAGCTCCACTACACAA | CACTGGCTACAAGCAGGCT |
| h-NRP1 | GGCGCTTTTCGCAACGATAAA | TCGCATTTTTCACTTGGGTGAT |
| h-EPAS1 | TTGCTCTGAAAACGAGTCCGA | GGTCACCACGGCAATGAAAC |
| h-fxyd5 | CTCACCATCGTTGGCCTGATT | TCCATGATAGTTGAGTCTGCTGA |
| h-col1a1 | GAGGGCCAAGACGAAGACATC | CAGATCACGTCATCGCACAAC |
| h-ACTA-2 | AAAAGACAGCTACGTGGGTGA | GCCATGTTCTATCGGGTACTTC |
| h-u-PA | CACGCAAGGGGAGATGAA | ACAGCATTTTGGTGGTGACTT |
| h-uPAR | TGTAAGACCAACGGGGATTGC | AGCCAGTCCGATAGCTCAGG |
| h-PAI-1 | ACCGCAACGTGGTTTTCTCA | TTGAATCCCATAGCTGCTTGAAT |
