# Supplementary material for: Endothelial senescence induced by PAI-1 promotes endometrial fibrosis
Source: Cell Death Discov. 2025 Mar 6;11:89. doi: 10.1038/s41420-025-02377-0 (PMC11885584; doi:10.1038/s41420-025-02377-0)
Supplement: Supplementary file 4 — Table S2. [file 41420_2025_2377_MOESM4_ESM.docx]

**Table S2 Antibodies used for immunohistochemistry (IHC), immunofluorescence (IF) and western blot (WB)**

| Primary antibodies | Catalog | Dilution | Application |
| --- | --- | --- | --- |
| CD31 | 66065-I-Ig | 1:1000 | IF |
| p21 | ab109520 | 1:200 | IF |
| p16 | 10883-1-AP | 1:200 | IF |
| IL-6 | 21865-1-AP | 1:200 | IF |
| CD68 | ab955 | 1:50 | IF |
| CD31 | ab28364 | 1:50 | IF |
| PAI-1 | ab222754 | 1:1000 | IF |
| uPAR | 17968-1-AP | 1:100 | IF |
| CD31 | 66065-2-Ig | 1:4000 | IF |
| p21 | 67362-1-Ig | 1:100 | IF |
| P21 | ab109520 | 1:2000 | WB |
| COL1A1 | ab6308 | 1:1000 | WB |
| α-SMA | ab7817 | 1:1000 | WB |
| IL-6 | 21865-1-AP | 1:1000 | WB |
| eNOS | cst 9586 | 1:1000 | WB |
| PAI-1 | ab222754 | 1:1000 | WB |
| uPAR | 17968-1-AP | 1:2000 | WB |
| u-PA | 17968-1-AP | 1:4000 | WB |
| p-smad2 | cst 8828 | 1:1000 | WB |
| smad2 | cst 3122 | 1:1000 | WB |
| α-SMA | ab7817 | 1:1000 | IHC |
| PAI-1 | ab222754 | 1:2000 | IHC |
| COL1A1 | 67288-1-Ig | 1:4000 | IHC |
